# Early initiations of first antenatal care visit and associated factor among mothers who gave birth in the last six months preceding birth in Bahir Dar Zuria Woreda North West Ethiopia

**DOI:** 10.1186/s12978-018-0646-9

**Published:** 2018-12-12

**Authors:** Yibeltal Alemu, Amanu Aragaw

**Affiliations:** Department of Reproductive Health, College of Medicine and Health Science, Bahir Dar University, P.O.Box 79, Bahir Dar, Ethiopia

**Keywords:** ANC, Maternal health, Pregnancy, Utilization, Ethiopia

## Abstract

**Background:**

Timing of Antenatal care booking is one of the basic components of antenatal care services; that helps to early detection, managing, and prevention of problems during the pregnancy and helps the mother to receive full packages of antenatal care services. However, in the world including Ethiopia, significant numbers of pregnant mothers were not booking the follow up on the recommended time. The main aim of this study was to assess the prevalence and factors that associated with the early timing of antenatal care visit in Bahir Dar Zuria District, North West Ethiopia.

**Methods:**

A community-based cross-sectional study was conducted. A total of 410 mothers have participated. Data were collected through the interview from March 1 to 30/2018 using a structured and pre-tested questionnaire. Data were clear, code, and enter into Epi-info version 7.1 and export to SPSS for farther analysis. Both bivariate and multivariate analyses were used. On bivariate analysis *p*-value, less than 0.2 were used to select the candidate variable for multivariate analysis. *P*-value and confidence interval were used to measure the level of significance on multivariate analysis and those variables whose *P*-value < 0.05 were considered as statically significant.

**Results:**

The prevalence of early timing of ANC in the study area was 46.8%; with [95% CI 40.5, 51.8]. Distances [AOR 2.47, 95% CI; 1.4, 4.2], Knowledge on the timing of ANC [AOR 2.1; 95% CI; 1.2, 3.7], No under-five children [AOR 2.7; 95% CI; 1.3, 5.8], having one under-five children [AOR 2.2; 95% CI; 1.1, 4.5], and wanted pregnancy [AOR 2.4, 95% CI, 1.3, 4.7] were affects the early timing of ANC.

**Conclusions:**

The prevalence of early timing of ANC was high when compared to the national figure and the Sub-Saharan country. Accessibility of health services, knowledge on the timing of ANC, under-five children, and desire for pregnancy were factors that affect the early timing of ANC.

## Plain English summary

Antenatal care is the care which provided for the mother during pregnancy to improve the health of the mother and unborn baby. World health organization is recommended for booking the services before the first trimester or 12 weeks of gestation. However, according to our country Ethiopia ministry health, it’s acceptable until 16 weeks of gestation. This study was trying to answer the level and associated factors through structured interview questionnaires.

The participants were asked their socio-demographic characteristics, reproductive characteristics, about antenatal care service utilization, and the challenges they face during antenatal care service utilization.

Of 400 participants 187 of the participants were initiated the follow-up before 16 weeks of gestation. This study reveals that the average month mothers were starting antenatal care visit at 16 weeks and 5 days. Short distances from the facilities, knowledge on the timing of ANC, not having under-five children, having one under-five child, and wanted or planned pregnancy was the main factor that affects the early timing of antenatal care visit.

In conclusion, the prevalence of early timing of ANC visit in the study area was high in comparison to the national figure. Factors that responsible for early initiation of ANC services were the accessibility of the services, knowledge about the timing of ANC, a desire of pregnancy, and under-five children. Policy action will be required to farther improve the timely booking of ANC.

## Background

Pregnancy is one of the most important periods in the life of a woman, a family, and a society [1]. While, because of these physiological changes that occur during pregnancy, mother and her baby were facing life treating problems. To avert the problem, there are different maternal health care services were provided for minimizing the problem. One of the services is Antenatal care (ANC) [2]. The aim of ANC service is to prevent health problems that affect both the fetus and mother and to ensure that each newborn child has a good start [3].

Nevertheless, true progress has been made globally in terms of increasing access and use, there are high numbers of maternal deaths and stillbirths were occurred [4]. Globally around 2.6 million stillbirths annually and 830 maternal deaths every day occur due to the pregnancy-related cause. Among all deaths, 99% occurred in the developing countries as compared as 1% in developed countries [5]. Many maternal and prenatal deaths occur in women who have not received timely, inadequate and no utilization of ANC [6]. Early antenatal care attendance during the first three months of gestation plays a major role in detecting and treating complications that occur during pregnancy [2].

The timing of Antenatal care booking is one of the basic components of ANC services. It helps to early detection, managing, and prevent problems that occur during the pregnancy time. According to WHO Focus antenatal care model recommendation, all pregnant mothers are better to start ANC booking within the first trimester of pregnancy (within 12 weeks) [3]. In addition, now a time World Health Organization adopt a new ANC model that recommend to increase the numbers of contact from four visits to eight contacts for the aim of reducing prenatal mortality and to improve women experiences of care [3, 7]. Actually, this new Antenatal care model still is not practicable in Ethiopia. However, according to the Federal Democratic Republic of Ethiopia Ministry of Health recommendation, the timing of ANC booking is acceptable until 16 weeks of gestation [8].

Existing evidence in the global shows that the prevalence of early timing of antenatal care visits were around 43%. According to the report, there is a high discrepancy between developed and developing regions [9]. The report showed that 85% of mothers in the developed region start their ANC follow up earlier but it was below 45% and less than 25% in the developing countries and sub-Sahara region respectively [10]. According to different demographic health survey report in sub-Sahara countries shows that the prevalence of early timing of ANC visit ranges from 17.6 to 20% [8, 11, 12].

According to Ethiopian demographic health survey, 2016 report indicates that 20% of pregnant mothers started their Antenatal care visit at the first trimester of pregnancy from that 44% of the mothers live in the urban, start ANC visit at the first trimester of pregnancy compared to 17% in the rural community [8]. Different literature conducted in Ethiopia also shows that the prevalence of early timing of ANC visit ranges from 17 to 41% [13–15].

Studies conducted in developing countries showed that residence, educational status of the mother, husband occupation, parity and planned or wanted pregnancy was factors for late ANC booking [16, 17]. Studies conducted in the urban Ethiopia shows that age of the mother, educational status of the mother, previous history of ANC, perceive adequacy of ANC, low monthly income, receiving advice on when to start ANC visits, household food insecurity, parity, urine test as a means of pregnancy recognition, and unplanned pregnancy was a determinant of the early timing of ANC visits [13, 14, 18–22].

Studies conducted in rural Ethiopia showed that age of the mother, parity, planned pregnancy, media access, knowledge about the time of ANC booking, and advised to book within 12 weeks was affected the timing of antenatal care visit [23, 24]. There are limited finding on the prevalence and factors associated with the early timing of ANC booking in the study area as well as in the country. For this situation, a cross-sectional study is important to address the gap. Therefore assessing the prevalence and factors that associated with early timings of antenatal care visit are important for setting intervention that helps to improve the utilization of ANC services.

## Methods and materials

### Study area

The study was conducted at Bahir Dar Zuria District; it is founded around Bahir Dar city administration 564 km far from the capital Addis. The District consists of 9 rural clusters. The total population of the District was estimated 220,410 from those, around 48.6% were female. Two thousand five hundred ninety-nine mothers in the district gave birth from August first to January last 2018; of that 86.4%, mothers have a history of ANC. In the District, there are 9 health centers, and 224 health professionals and 32 health extension workers were enrolled [25].

### Study design and period

A Community-based cross-sectional study was conducted from March 1 to 30, 2018.

### Source populations

All mothers who have a history of ANC and gave birth in the last six months in Bahir Dar Zuria district.

### Study population

Those selected mothers who have a history of ANC and gave birth in the study in the last six months during data collection period in Bahir Dar Zuria district.

#### Inclusion criteria

All mothers who have a history of ANC and gave live birth.

#### Exclusion criteria

Women excluded from the study were those who are not hearing and seriously ill during the data collection period.

### Sample size determination

The sample size for the study was calculated using a single population proportion formula with the following assumptions. Based on findings from a previous study conducted in the central zone of Tigray (Ethiopia) [26]. The proportion of women who start ANC before the fourth month was found to be 41% By assuming a margin of error of 5%, *Z*_*α*/2_ = value for 95% CI (1.96), proportion = 41%, and the non-response rate of 10%, a sample size of 410 women obtained.

### Sampling procedures and technique

All 9 clusters in the study area were included. The health extension workers immunization registration book was used to easily identify mothers who gave birth for the last six months in the District and whether they have a history of ANC from August 1 to January 30, 2018, G.C. Subsequently, the lists of the entire mother who have history ANC were found from the health extension worker registration books. Then the simple random sampling technique was used to select the study participants from those who have a history of ANC.

### Data collection tools and procedures

Data were collected using a structured questionnaire which adopted and modified from Ethiopian DHS and other previous studies. The questionnaire was prepared in English and it translated into Amharic then translated back to English to check for consistency.

Data were collected via interview. Seven diploma holder nurses were recruited for data collection, 3 supervisor degree holder nurses were assigned for supervisory activities along with the principal investigator.

### Data quality controls

The prepare questionnaire and tools were translated into Amharic and then translated back to English for consistency. The Amharic version of the questionnaire was pre-test among 41(10%) of the sample population in the Cluster not include in the study. Training was provided for the data collectors and supervisors on the objectives, relevance of the study, how to keep the confidentiality of the information and techniques of interviews for two consecutive days. The supervisors have supervised the data collection process every day and the principal investigator also cheeks the collected questionnaire for completeness every other day.

### Data processing and analysis

Data were cleaned, code and entered into Epi-info version 7.1 then exported to SPSS version 23 for analysis. Descriptive analysis was carried out to see the distribution of independent variables. Binary logistic regression was used to examine associations between the dependent variable and each independent variable. Based on the bivariate analysis those factors whose crude associations to the timing of antenatal care booking at *p* < 0.2 was entered into the multivariate analysis to get adjusted odds ratio.

The strength of association was determined by using a crude odds ratio in the bivariate analysis and adjusted odds ratio in multivariate analysis. *P*-values and 95% confidence interval was used to determine the level of significance of the association. *P* < 0.05 considered as statistically significant. Hosmer and Lemeshow Test were used for checking the model fitness of logistic regressions.

## Results

Out of 410 mothers who have a history of Antenatal care follow up in Bahir Dar Zuria District, 400 complete the interview administer questionnaires. Therefore the data analysis was made based on 400 respondents that have been completed the interview. The response rate was 97.56%.

### Socio-cultural characteristics

Among the total participants, one hundred twenty-eight (32%) of the respondents were in the age group of 25-29 years. The mean ages of the respondents were 28.47 (SD ± 5.47). Three hundred ninety-four (98.5%) of the respondents were followers of Orthodox religion the rest were Muslim. Three hundred ninety-eight (99.5%) of the respondents were Amhara ethnicity. Three hundred-ninety (97.6%) of the respondents were married. Fifty (12.5%) of the respondents were attended formal education.

Eighty-four (21%) and 85 (21.5%) of the household were rich and richest wealth index respectively. Two hundred sixty-two (65.5%) of the respondents were poor household decision making power. One hundred fourteen (28.5%) of the respondent traveling more than one hour to reach the ANC services. The median time travel to reach the ANC services was 60 min. Three hundred sixty-seven (91.8%) of the respondents were traveling on foot. (Table 1).

**Table 1:** The socio- cultural characteristics of the mother in Bahir Dar Zuria district North West Ethiopia 2018(*n* = 400)

Variables	Frequency	Percent (%)
Age		
< 20 years	9	2.3
20–24 years	89	22.2
25–29 years	128	32
30–34 years	113	28.3
≥35 years	61	15.2
Educational status of the mother		
Not attained formal education	350	87.5
Primary level (Grade 1to 8)	41	10.3
Secondary (Grade 9 to 12)	9	2.2
Marital status		
Single	6	1.5
Married	390	97.6
Divorced	3	0.8
Widowed	1	0.3
Occupation of the mother		
Farmer	384	96
Trader	12	3
Student	3	0.75
Governmental employer	1	0.25
Wealth index		
Poorest	67	16.75
Poor	74	18.5
Middle	90	22.5
Rich	84	21
Richest	85	21.25
Accompanion during ANC visit		
Yes	267	66.8
No	133	33.3
Family size		
1–3	89	22.2
4–5	142	35.5
Above 5	169	42.3
Numbers of under five children		
No under five	108	27
One under five	234	58.5
Two under five	58	14.5
Distance		
Less than 30 min	146	36.5
30 min to one hour	140	35
More than one hour	114	28.5

### Reproductive history

Three hundred twenty-two (80.2%) of the respondent were knowledgeable about the timing of antenatal care. Two hundred-thirteen (53.3%) of the respondents were multi-gravid. Two hundred twenty-five (56.3%) of the respondents were multi-Para. Fifty-six (14%) of the participants has had an unwanted pregnancy. (Table 2.

**Table 2:** reproductive characteristics of the mother in Bahir Dar Zuria district North West Ethiopia 2018(n = 400)

Variable	Frequency	Percent
Numbers of pregnancy		
One	80	20
2 to 5	213	53.2
≥ 5	107	26.8
Parity		
Primipara	91	22.7
Multi-para	225	56.3
Grand multi-para	84	21
Knowledge of the mother on the timing of ANC		
Knowledgeable	322	80.5
Not knowledgeable	88	19.5

### Prevalence of early timing of antenatal care booking

**Fig. 1:**
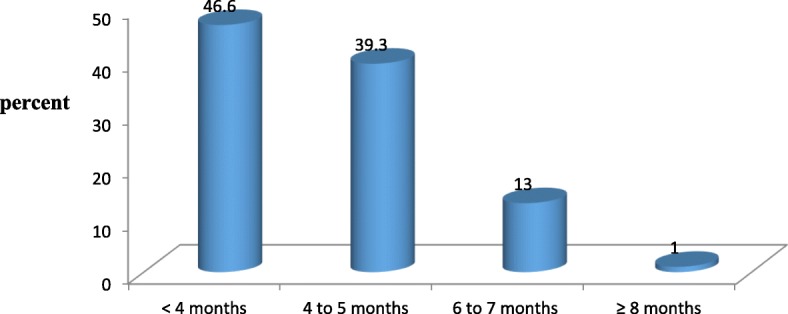
Timing of first ANC booking among ANC utilized mothers in Bahir Dar Zuria District North West Ethiopia 2018. The figure showed that among all participants, 46.7% of the respondents, were booking there first ANC visit before four months of pregnancy and 39.3% of the respondents were started the follow up at four to five months. In addition, 13 and 1% of the respondents were booking there first ANC at six to seven; and eight and after months respectively

Among the participants one hundred eight-seven (46.7%) with 95% CI [40.5, 51.8] of the respondents, were booking there first ANC visit before four months of pregnancy. The average months of starting the follow up were 16 weeks and 5 days. One hundred fifty-seven (39.3%) of the respondents were started the follow up at four to five months. (Fig. 1).

### Factors that associated with early initiations first antenatal care visit

In the bivariate analysis, family size, decision-making power, distance, wealth index, knowledge, under five, age, accompanying person, urine test, a desire of pregnancy were candidate variable for multivariate analysis. And on multivariate analysis, distances, knowledge, under-five children, and desire for pregnancy were true determinants of early time antenatal care visit at the *p*-value less than 0.05.

The factors that directly affect the early timings of Antenatal care visit were short distances from the facility, knowledge on the timing of ANC, not having under-five children, having one under-five child, and wanted or planned pregnancy were factors that affect the early timing of antenatal care visit.

The odds of early timing of ANC visit among mothers who travel less than 30 min to reach ANC services were 2.47 times higher than those who travel more than one hour [AOR 2.47, 95% CI; 1.4,4.2]. The odds of early timing of antenatal care visit among mothers who were knowledgeable on the timing of ANC visit were 2.1 times higher than the counterparts [AOR 2.1; 95% CI; 1.2, 3.7]. The odds of early timing of ANC visit among mothers who have no under five children 2.7 and one under five children 2.2 times higher than those who had two under-five children [AOR 2.7; 95% CI; 1.3,5.8], [AOR 2.2; 95% CI; 1.1,4.5] respectively. In addition to this, the odds of early timing of ANC visit among mothers with wanted pregnancy were 2.4 times higher than the counterparts [AOR 2.4, 95% CI, 1.3, 4.7]. (Table 3).

**Table 3:** Bivariate and multivariable association of factors, with the timing of ANC initiation in Bahir Dar Zuria District, North West Ethiopia 2018. (n = 400)

Variable	Early ANC initiation		COR (95% CI)	AOR (95% CI)	*p*-value
	Yes	No			
Distance					
Less than 30 min	70	44	3(1.9,5.3)	2.47(1.4,4.2)*	0.002
30 min to 1 h	70	76	1.8(1,2.9)	1.6(0.98,2.6)	0.71
More than 1 h	47	93	1	1	
Accompanying person					
Yes	135	132	1.59(1,2.4)	1.3(0.86,2.2)	
No	52	81	1	1	
Family size					
< 5	92	71	1.94(1.3,2.9)	1.4(0.62, 2.1)	
≥ 5	95	142	1	1	
Knowledge on ANC					
Knowledgeable	165	157	2.67(1.5,4.5)	2.1(1.2,3.7)*	0.016
Not knowledgeable	22	56	1	1	
Numbers of under five children					
No under five	58	50	4 (1.9,8.3)	2.7(1.3,5.8)*	0.014
One under five	116	118	3.4 (1.6, 6.6)	2.2(1.1,4.5)*	0.030
Two under five	13	45	1	1	
Age of the mother					
≤ 24 years	58	40	2.39(1.2,4.6)	1.7(0.85,3.5	
25 to 34	106	135	1.29(0.7,2.3)	1.2(0.66, 2.25)	
≥ 35	23	38	1	1	
Wanted or planned pregnancy					
Yes	173	171	3(1.6,5.7)	2.4(1.3,4.7)*	0.008
No	14	42	1	1	

## Discussion

The prevalence of early timing of ANC visits was 46.8%. The factors that directly affect the early timing of ANC visits were distances, knowledge of the mother on the timing of ANC, numbers of under-five children, and desire of pregnancy.

This finding shows that the prevalence of early first time antenatal care visits was 46.8 with 95% CI [40.5, 51.8] which is in line with the prior study conduct in Gonder Ethiopia 47.4% [18], and central Tigray Ethiopia 41% [14]. However the finding of this study was lower than study conduct in Addis Ababa Ethiopia 58% [27], and Nepal 70% [17]. The difference might be due to the study area in Addis Ababa there are accessible and available services and more aware of the existing services than the study area.

The finding of this study was higher than study conduct in Gedeo Zone Ethiopia 35.4% [20], Ethiopian DHS 20% [8], and in the Global estimate 24% [10]. This inconsistency could be attributed to the scope of the study, the fact that EDHS covered more remote. It is also significant to note down the time gap between the EDHS and this study.

This study also shows that mothers who live within short distances from the health facility were more likely early initiate ANC visit than those who travel more than one hour. This finding was in line with the finding of previous study conduct in Cameron [28]. This might be due to women live in a developing country spent more times for activities like drawing water, household chores, rearing children and for agricultural activities rather than their health. As a result, they didn’t think to attain the health facility for receiving ANC services because they have no time to go there. In addition to this, it may impose them for extra costs for transportation as well as loss from agricultural production.

This study also explains that mothers who have had knowledgeable on the timing of ANC were more likely to early initiate the ANC services than those not knowledgeable. This finding was consistent in meta-analysis study conduct in Ethiopia [29], and Southern Ethiopia [30, 31] this might be due to knowledge is important to realize the existing services and the timing of initiating ANC visits. In addition to this, high illiteracy level in the community may cause poor knowledge in the community.

Mothers who want her pregnancy were more likely to start the follow up early than the counterpart. This finding was in line with previous study conduct in Debre Markos Ethiopia [32], Sothern Ethiopia [33], Ethiopia [29], Tigray Ethiopia [34], South Africa [35], and Nepal [17]. This might be due to when the mother prefers the pregnancy she is eager to keep the health of the baby. Due to that, they are excited to attain the follow up earlier.

In addition in this study the odds of early initiations of ANC visit among women who have no under-five children 2.7 and one under-five children 2.2 times higher than those who had two under-five children [AOR 2.7; 95% CI; 1.3,5.8], [AOR 2.2; 95% CI; 1.1,4.5] respectively. There are limited finding with regard to the relation between timing of initiation and numbers of under-five children in the previous study but in this study numbers of under-five children was one of the determinant factors for the timing of initiation of ANC. This might be due to women may responsible for caring for their baby.

## Conclusion

The prevalence of early initiation of ANC in the study area was high in comparison to the national prevalence of the country. The factor that responsible for early initiation of ANC services were distance, knowledge on the timing of ANC, a desire of pregnancy, and less and family size.

## Recommendation

For federal and regional health bureau to increase the awareness of timings of antenatal care utilization and increase access and availability of family planning method for preventing unintended pregnancy.

### Limitation

The finding of this study was not triangulated on qualitative finding.

## Abbreviations

ANC: Antenatal Care
DHS: Demographic Health Survey
E.C: Ethiopian Calendar
EDHS: Ethiopian Demographic Health Survey
FANC: Focus Antenatal Care
Km: Kilometer
SDG: Sustainable Development Goals
SPSS: Statistical Package for Social Sciences Software
UN: United Nation
UNICEF: United Nations International Children’s Emergency Fund
USAID: United State Aid for International Development
WHO: World Health Organization
